# Supplementary material for: Evaluation of the effect of chickenpox vaccination on shingles epidemiology using agent-based modeling
Source: PeerJ. 2018 Jun 20;6:e5012. doi: 10.7717/peerj.5012 (PMC6015493; doi:10.7717/peerj.5012)
Supplement: Supplemental Information 1 [file peerj-06-5012-s001.pdf]

Supplementary Material for

Evaluation of the effect of chickenpox vaccination on shingles epidemiology using agent-based modeling.

Table of contents

1. Supplementary Figure 1: Equations to calculate the shingles immunity waning timer.
2. Supplementary Figure 2: An example of the space dependence represented in a distance-based network.
3. Supplementary Table 1: Data extracted from sources for the calibration and fitting of the model.
4. Supplementary Figure 3: Age-specific proportion of the population with varicella antibody.
5. Supplementary Figure 4: Chickenpox and shingles incidence between urban and rural communities, both before and after vaccination.
6. Supplementary Figure 5: Simulated and empirical age-specific shingles incidence rate for scenarios that met calibration with various duration of boosting and waning of immunity rates at time 0.
7. Supplementary Figure 6: Simulated and empirical age-specific shingles incidence rate for scenarios that did not met calibration with various duration of boosting and waning of immunity rates at time 0.
8. Supplementary Figure 7: Simulated and empirical age-specific chickenpox incidence for different duration of boosting and waning of immunity, all scenarios that met calibration at time 0.
9. Supplementary Figure 8: Number of shingles cases by age at time 10, 25, 50 and 7 by experiment.
10. Supplementary Figure 9: Age distribution of shingles cases in baseline scenario.
11. Supplementary Figure 10: Frequency of connections between urban and rural agents.

Shingles Immunity Waning Timer = (1) Years Protected Through Infection + (2) Years Protected Through Boosting

(1) Years Protected Through Infection = $\frac{\min\,(0,\log(forceOfReactivation/InitialCMI)}{\min(0,\log(1-waningOfImmunityRateShingles)}$

(2) Years Protected Through Boosting= Number of times an agent comes into significant contact with VZV (in the form of chickenpox or shingles) X The duration in years of each boosting event

Where:

ForceOfReactivation= The strength of shingles reactivation, i.e. the amount of VZV-CMI need to stop reactivation in the form of shingles, the value for each individual in our population is drawn from a gamma distribution.

InitialCMI= The initial level of VZV-CMI protection conferred following chickenpox, the value for each individual in our population is drawn from a normal distribution.

WaningOfImmunityRateShingles= the rate of annual loss of VZV protection (1/years). The WaningOfImmunityRateShingles is a fixed rate that can be altered in our model using the waning of immunity coefficient.

Duration of each boosting event= The number of years of protection gained through each significant boosting event, this value is based on calibration results.

This equation was derived from the model presented in Ogunjimi *et al*.[25]

Supplementary Figure 1: Equations to calculate the shingles immunity waning timer.

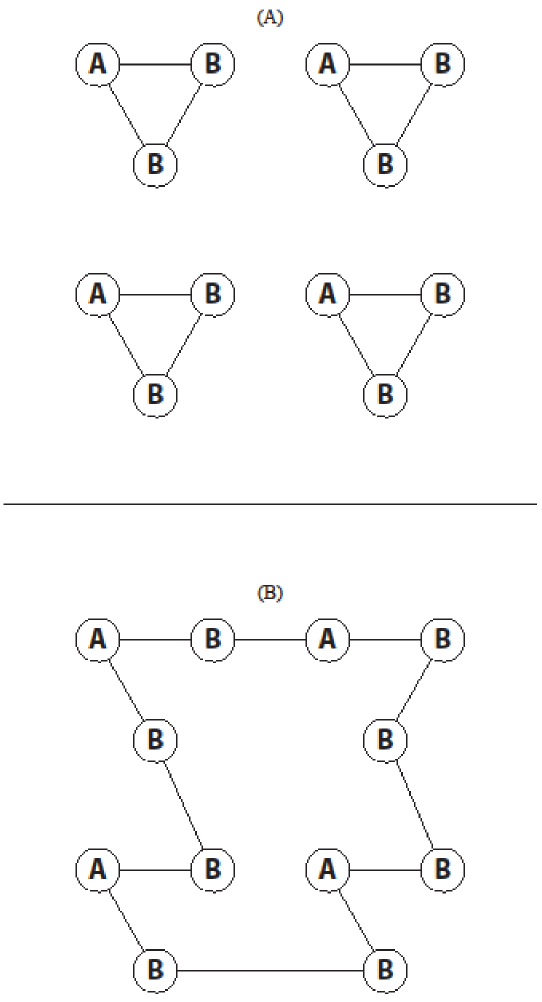

Consider the two networks shown in the above figure Agents are divided into two age groups, A and B. The table below shows the count of contacts between each of the age groups. This table applies to both networks, even though their arrangement is different. In Figure S2 (A), an infection in age group A in the first quadrant can potentially spread to two other people. In Figure S2 (B), an infection of the same person can potentially spread to the entire population. This demonstrates spatial dependence that is captured by an ABM but not evident when summarizing the network structure in the form of a contact matrix.

Age Group	**A**	**B**
**A**	0	8
**B**	8	8

Supplementary Figure 2: An example of the space dependence represented in a distance-based network.

.

Supplementary Table 1: Data and references used in the calibration and fitting of the model.

**Model data calibrated**	**Source outcomes that we compared to model data (by age group)**	**Years of data collection**	**Source**
Age-specific shingles rates of infection (Parameters varied: Duration of boosting, waning of immunity coefficient, exogenous infection rate, and shingles connection range)	Fig 3 “Age-specific medically attended shingles rates, per 1000 population, and availability of chickenpox vaccine: 1994-98 (pre-licensure)” - Medically attended (ages: <1, 1-4, 5-9, 10-19, 20-24, 25-29, 30-34, 35-39, 40-44, 45-49, 50-54, 55-59, 60-64, 65-69, 70-74, 75-79, 80-84, 85-89, 90+)	1994-1998	Russell et al. (2014)[4]
Age-specific chickenpox rates of infection (parameters varied: exogenous infection rate, shingles connection range, underreporting factor)	Table 2 “Mean annual rates of varicella-related outcomes during pre-vaccine (FY1992-1998)” - Office visits (ages: <1, 1-4, 5-9, 10-19, 20-49 and 50+)	1992-1998	Kwong et al. (2008)[27]

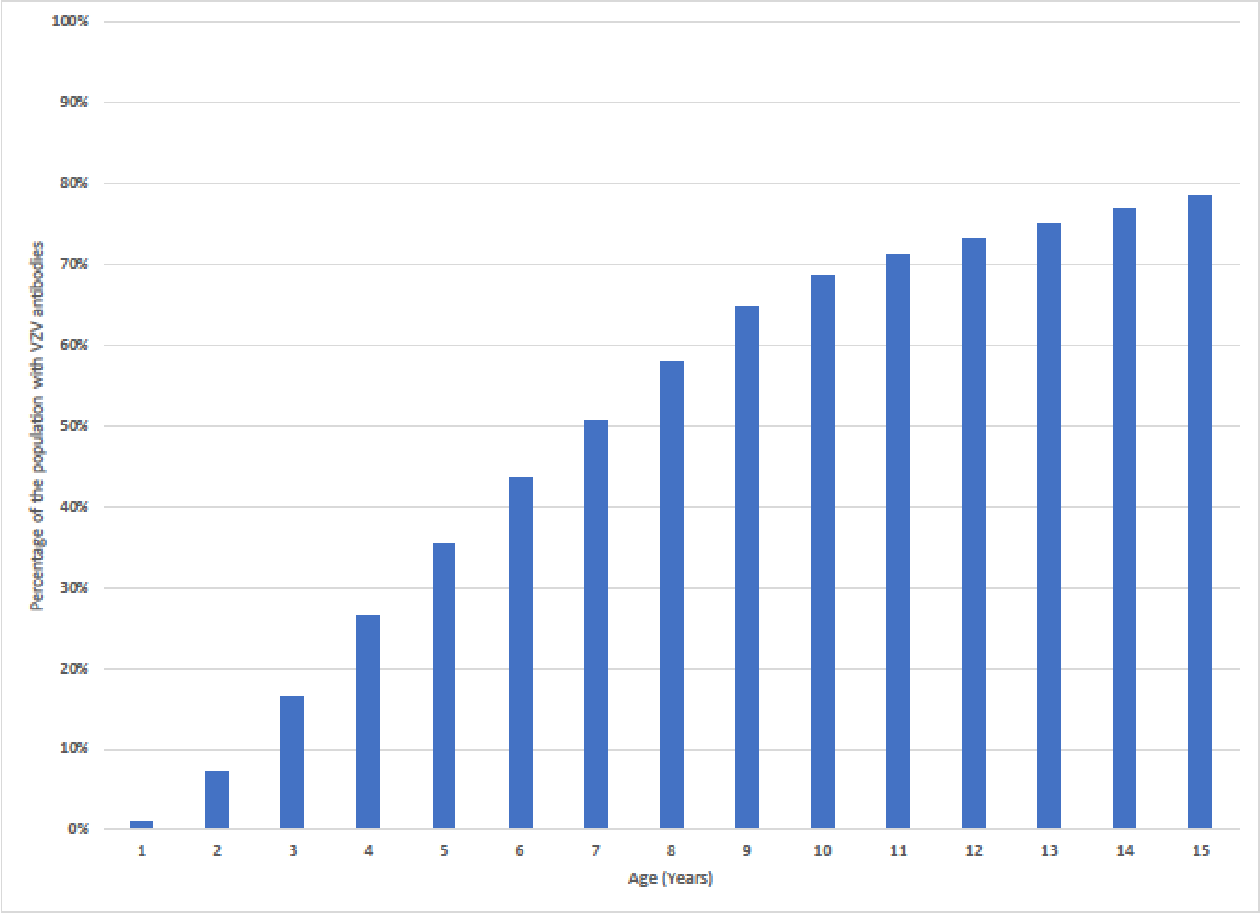

Supplementary Figure 3: Age-specific proportion of the population with varicella antibody.

(A)

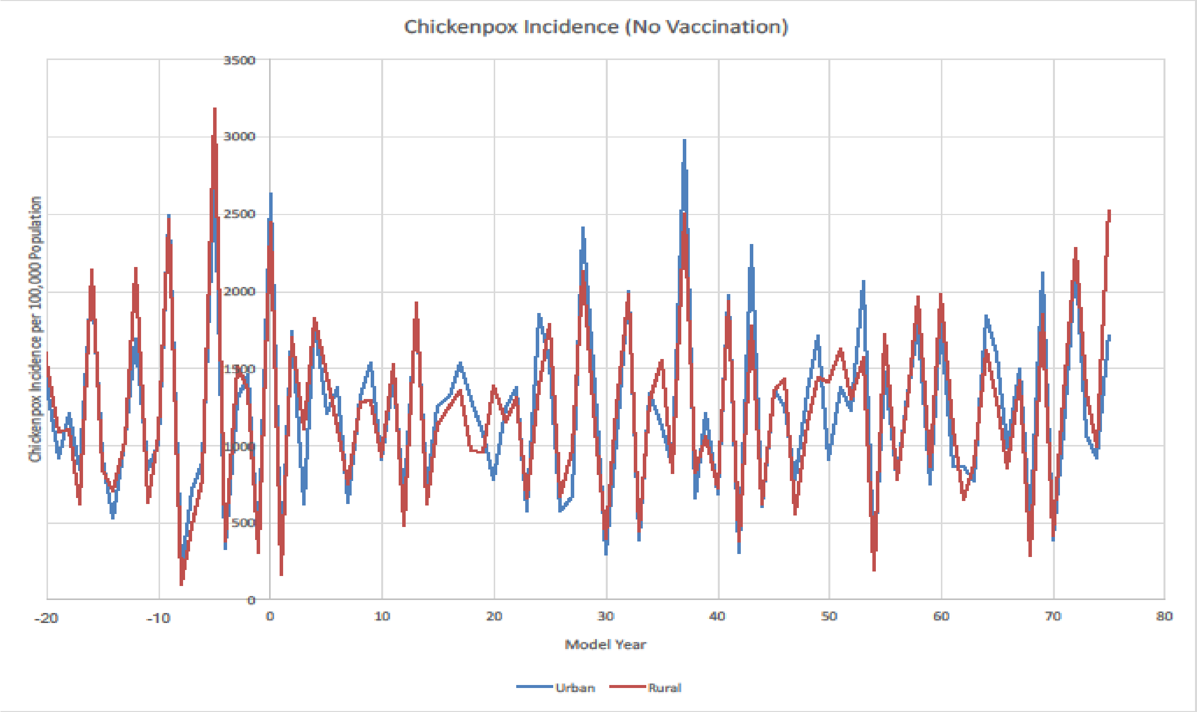
Chickenpox Incidence (No Vaccination)
Chickenpox Incidence per 100,000 Population
3500
3000
2500
2000
1500
1000
500
0
-20
-10
0
10
20
30
40
50
60
70
80
Model Year
Urban
Rural

(B)

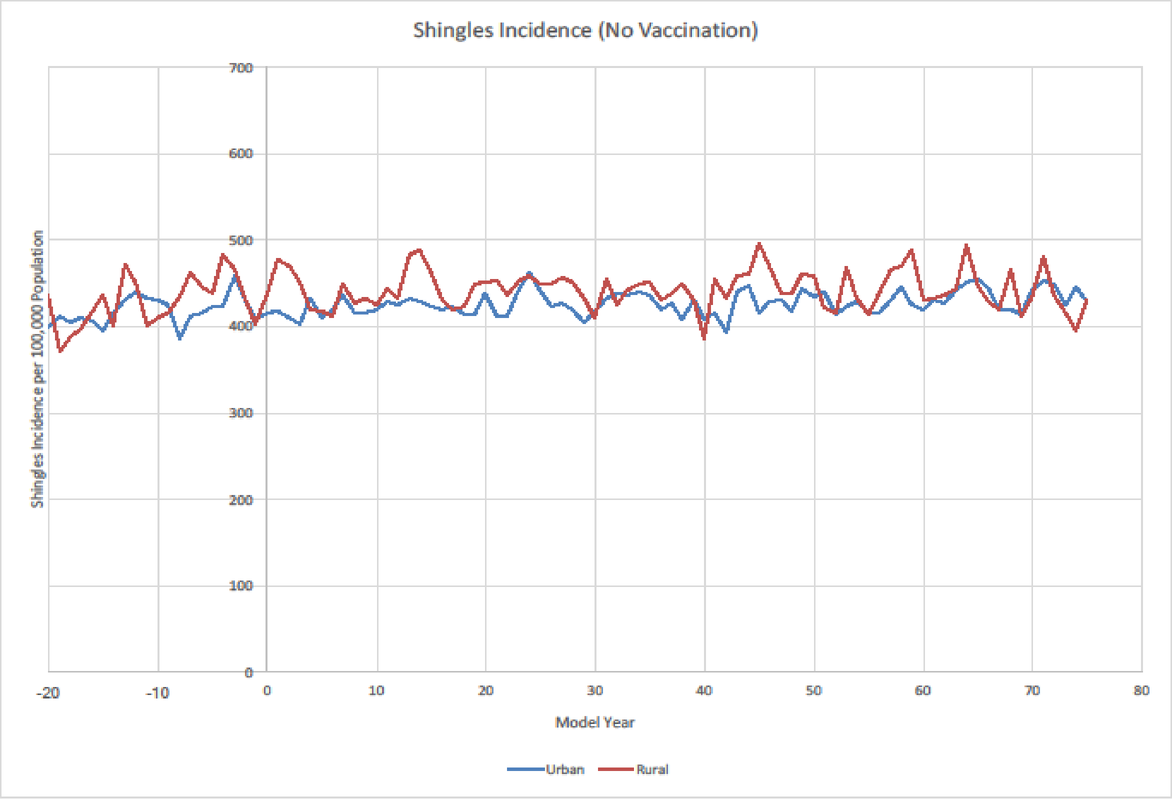
Shingles Incidence (No Vaccination)
Shingles Incidence per 100,000 Population
700
600
500
400
300
200
100
0
-20
-10
0
10
20
30
40
50
60
70
80
Model Year
Urban
Rural

(C)

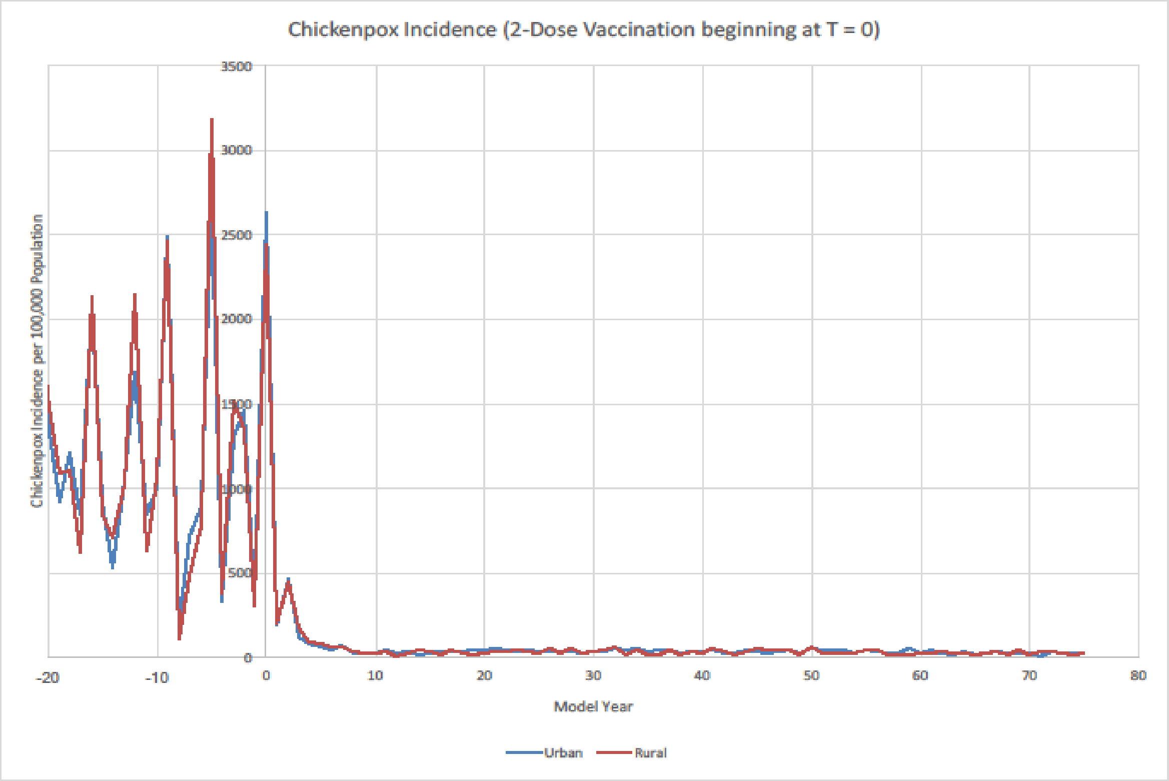

(D)

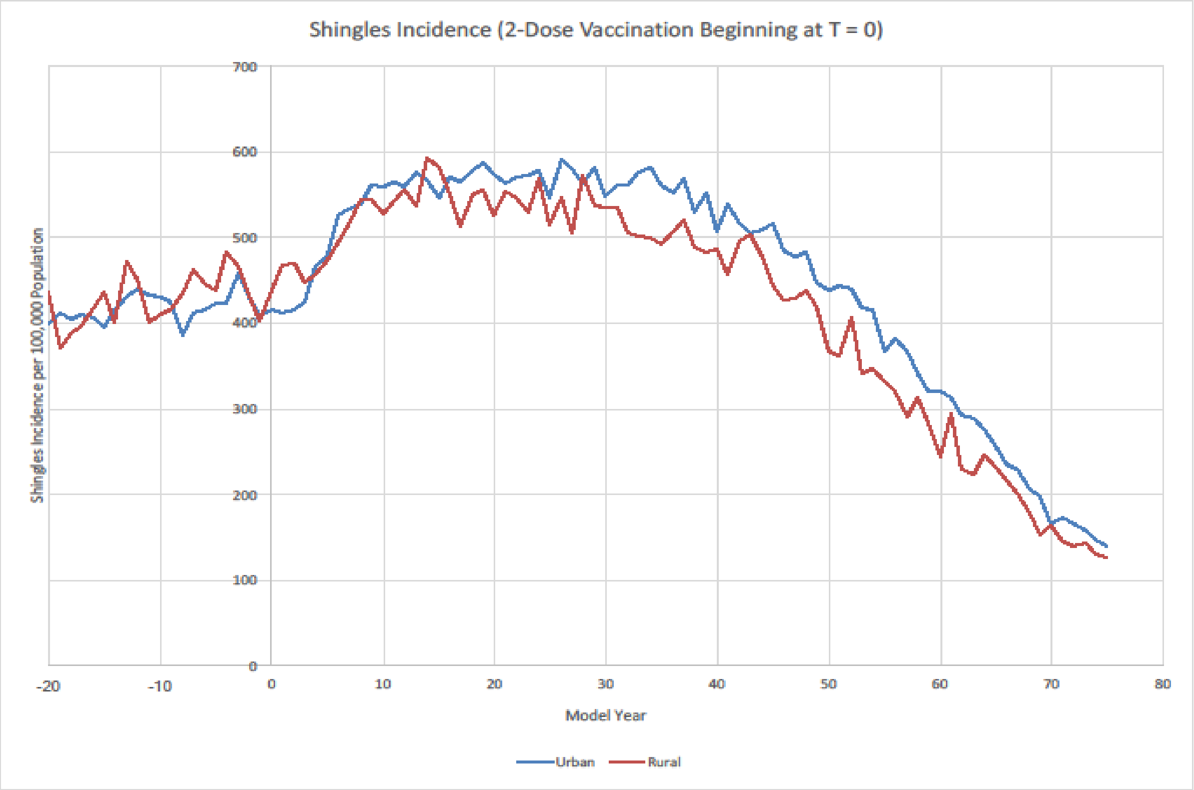

Supplementary Figure 4: Outcomes by urban and rural communities. (A) Chickenpox incidence pre-vaccination. (B) Shingles incidence pre-vaccination. (C) Chickenpox incidence post-vaccination. (D) Shingles incidence post-vaccination.

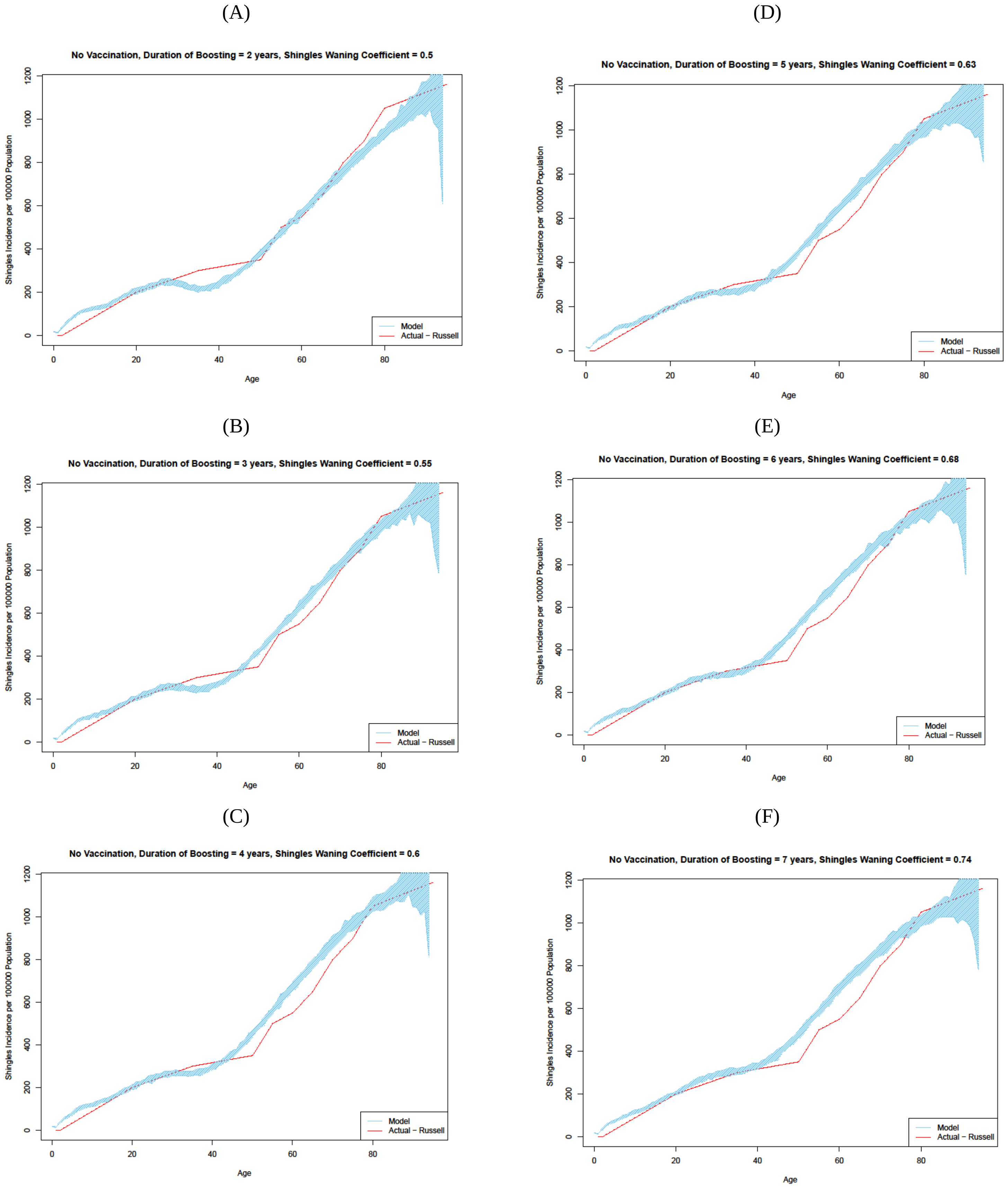

Supplementary Figure 5: Simulated and empirical age-specific shingles incidence rate for scenarios that met calibration with various duration of boostings and waning of immunity rates at time 0. (A) DoB= 2 years, WoI= 0.5. (B) DoB= 3 years, WoI= 0.55. (C) DoB= 4 years, WoI= 0.6. (D) DoB= 5 years, WoI= 0.63. (E) DoB= 6 years, WoI= 0.68. (F) DoB= 7 years, WoI= 0.74. Blue polygons represent the min and max of the 30 simulated runs.

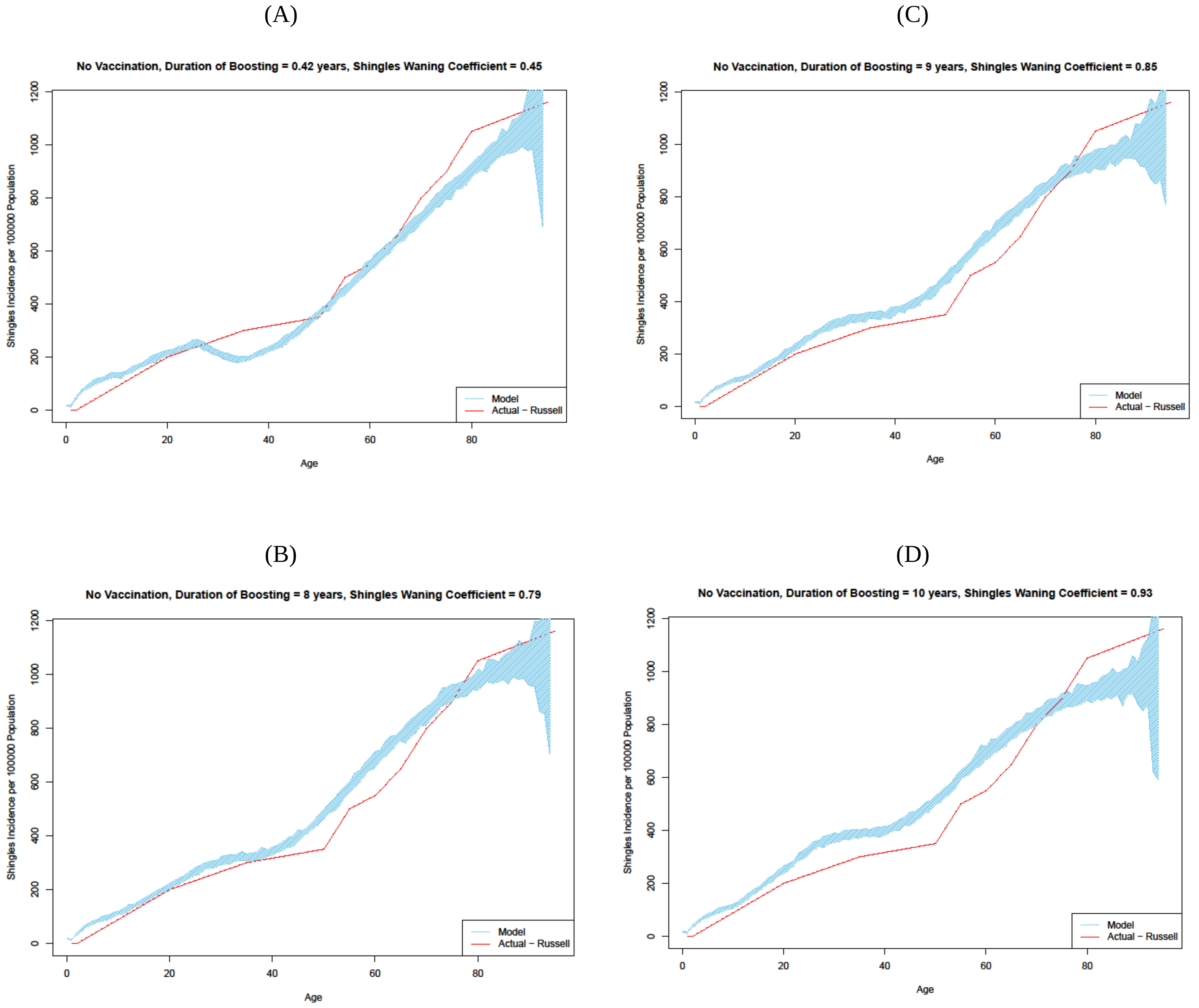

Supplementary Figure 6: Simulated and empirical age-specific shingles incidence rate for scenarios that did not met calibration with various duration of boostings and waning of immunity rates at time 0. (A) DoB= 0.42 years, WoI= 0.45. (B) DoB= 8 years, WoI= 0.79. (C) DoB= 9 years, WoI= 0.85. (D) DoB= 10 years, WoI= 0.93. Blue polygons represent the min and max of the 30 simulated runs.

(A)

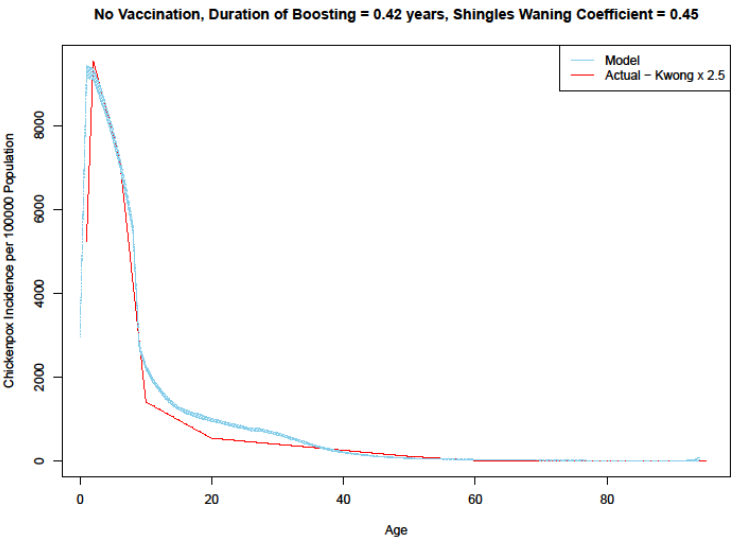
No Vaccination, Duration of Boosting = 0.42 years, Shingles Waning Coefficient = 0.45
Model
Actual − Kwong x 2.5
Chickenpox Incidence per 100000 Population
Age
0
2000
4000
6000
8000
0
20
40
60
80

(B)

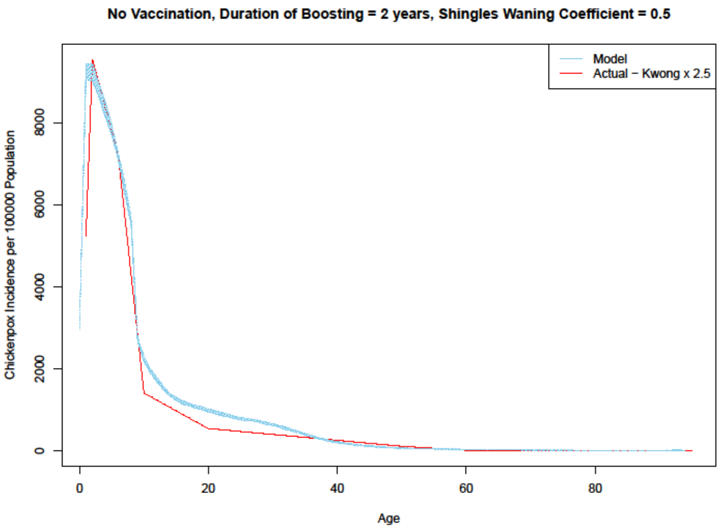
No Vaccination, Duration of Boosting = 2 years, Shingles Waning Coefficient = 0.5
Model
Actual − Kwong x 2.5
Chickenpox Incidence per 100000 Population
Age
0
2000
4000
6000
8000
0
20
40
60
80

(C)

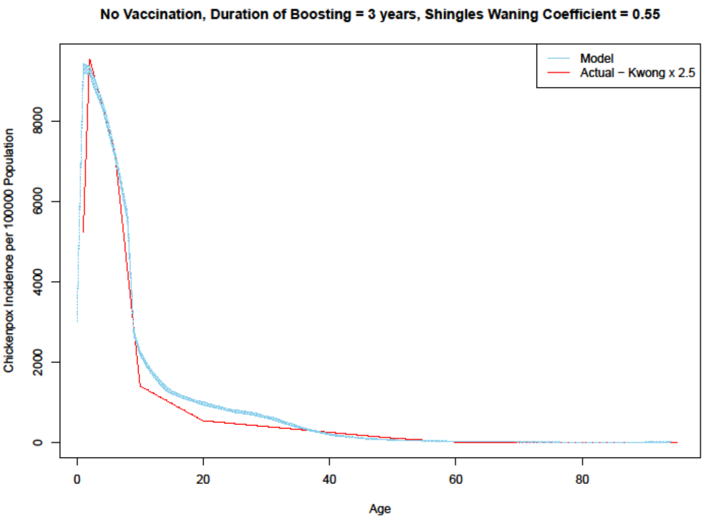
No Vaccination, Duration of Boosting = 3 years, Shingles Waning Coefficient = 0.55
Model
Actual − Kwong x 2.5
Chickenpox Incidence per 100000 Population
Age
0
2000
4000
6000
8000
0
20
40
60
80

(D)

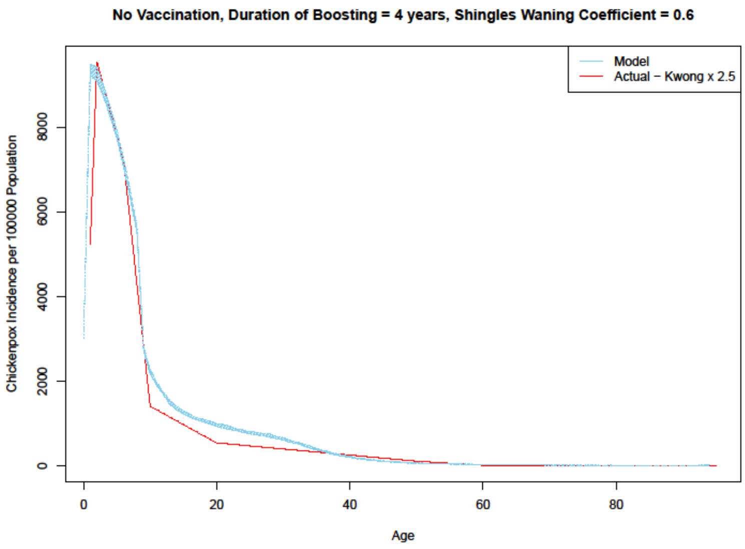
No Vaccination, Duration of Boosting = 4 years, Shingles Waning Coefficient = 0.6
Model
Actual − Kwong x 2.5
Chickenpox Incidence per 100000 Population
Age
0
2000
4000
6000
8000
0
20
40
60
80

(E)

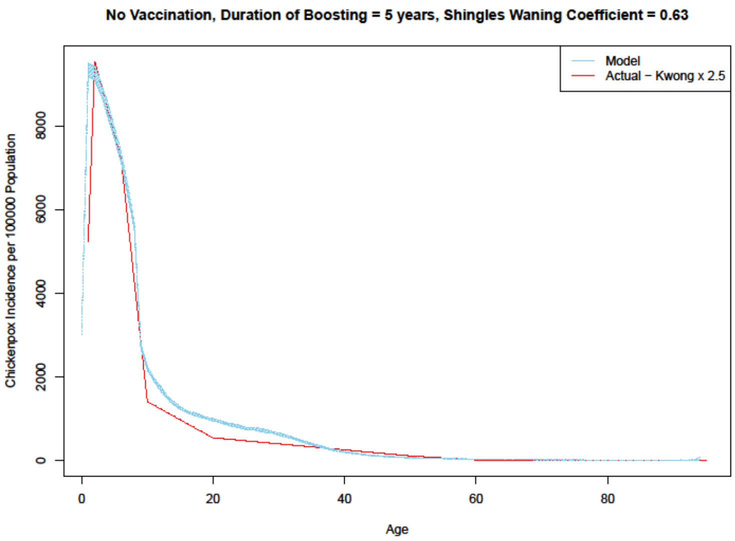
No Vaccination, Duration of Boosting = 5 years, Shingles Waning Coefficient = 0.63
Model
Actual − Kwong x 2.5
Chickenpox Incidence per 100000 Population
Age
0
2000
4000
6000
8000
0
20
40
60
80

(F)

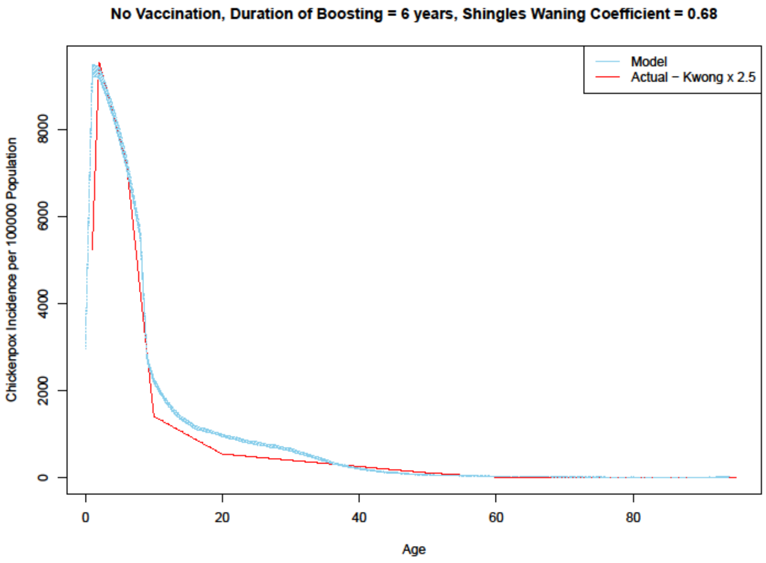
No Vaccination, Duration of Boosting = 6 years, Shingles Waning Coefficient = 0.68
Model
Actual − Kwong x 2.5
Chickenpox Incidence per 100000 Population
Age
0
2000
4000
6000
8000
0
20
40
60
80

(G)

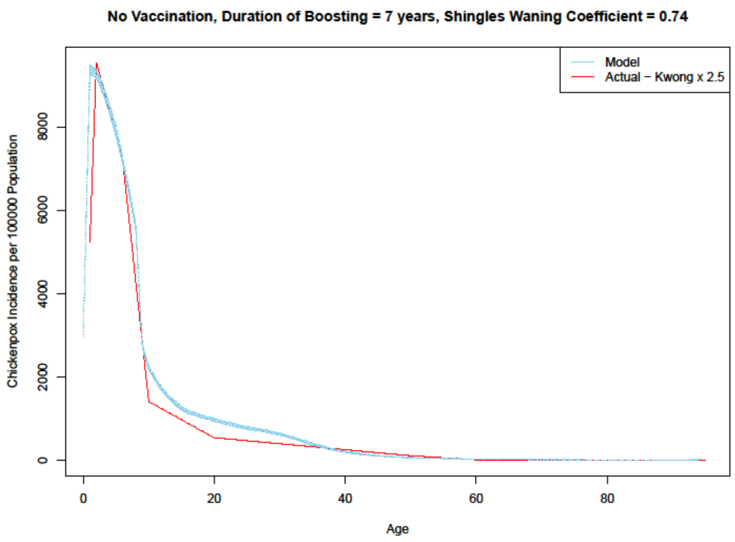

(I)

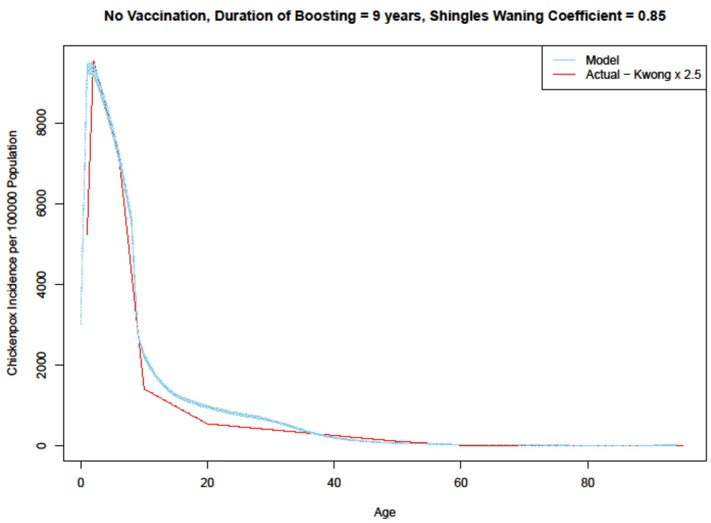

(H)

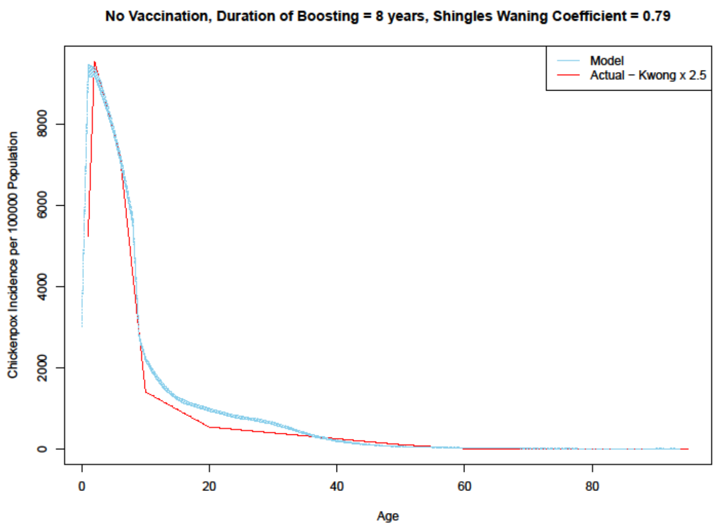

(J)

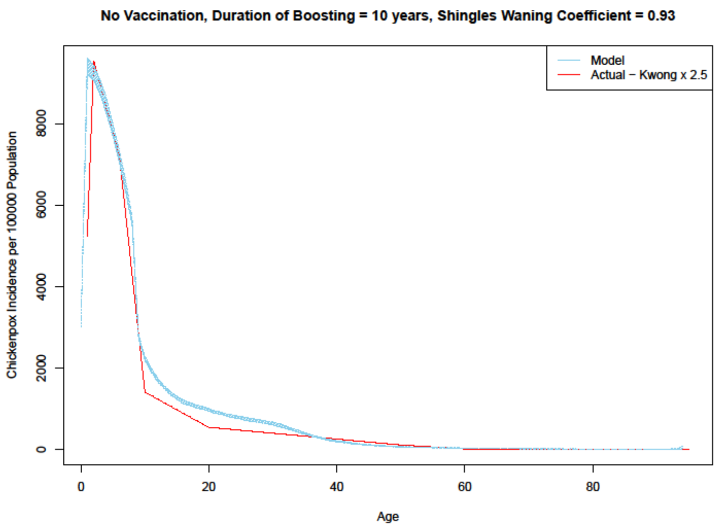

Supplementary Figure 7: Simulated and empirical age-specific chickenpox incidence for different duration of boosting and waning of immunity, all scenarios that met calibration at time 0. (A) DoB= 0.42 years, WoI= 0.45. (B) DoB= 2 years, WoI= 0.5. (C) DoB= 3 years, WoI= 0.55. (D) DoB= 4 years, WoI= 0.6. (E) DoB= 5 years, WoI= 0.63. (F) DoB= 6 years, WoI= 0.68. (G) DoB= 7 years, WoI= 0.74. (H) DoB= 8 years, WoI= 0.79. (I) DoB= 9 years, WoI= 0.85. (J) DoB= 10 years, WoI= 0.93. Blue polygons represent the min and max of the 30 simulated runs.

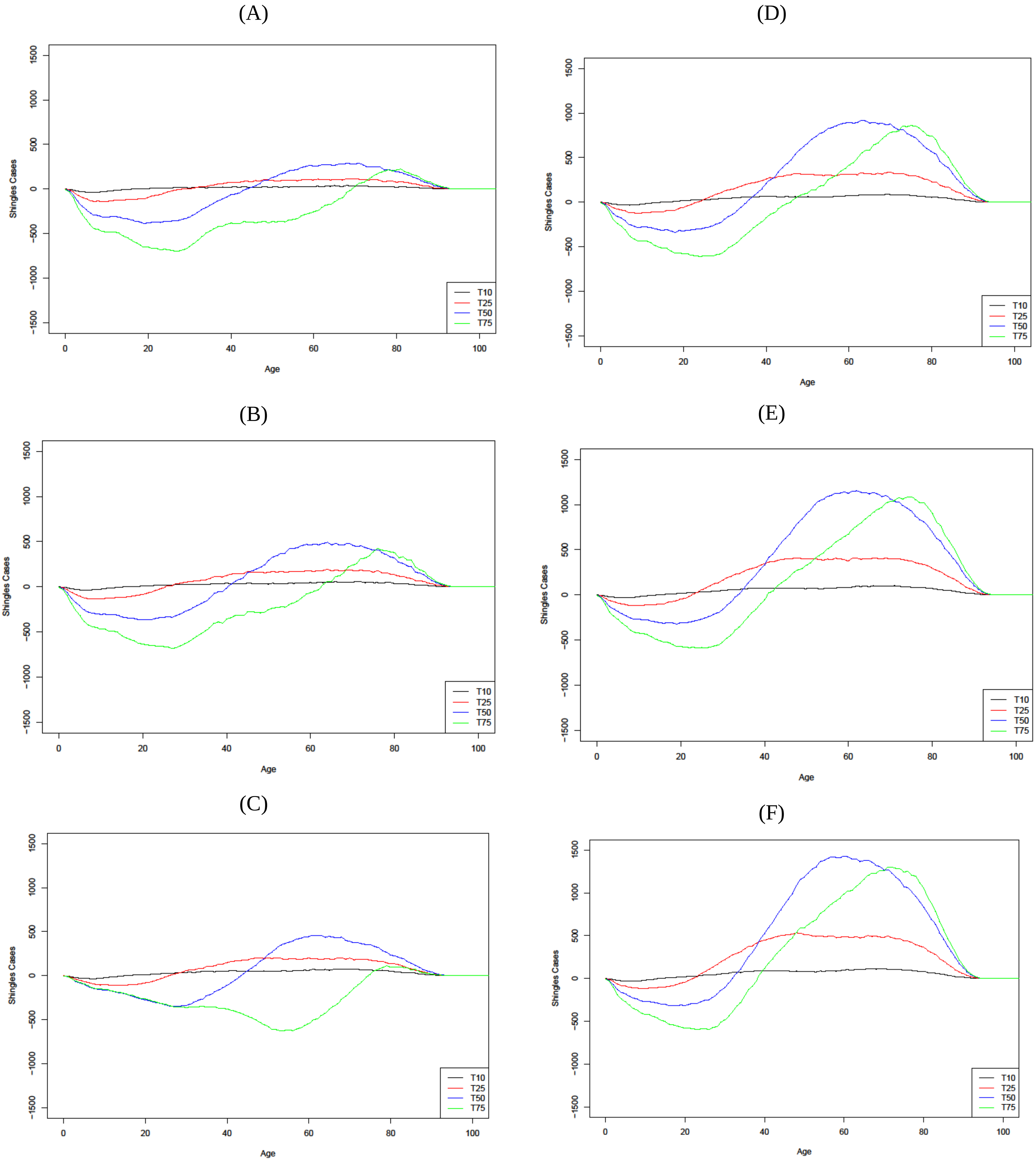

Supplementary Figure 8: Number of shingles cases by age at time 10, 25, 50 and 75 years by scenario. (A) DoB= 2 years, WoI= 0.5. (B) DoB= 3 years, WoI= 0.55. (C) DoB= 4 years, WoI= 0.6. (D) DoB= 5 years, WoI= 0.63. (E) DoB= 6 years, WoI= 0.68. (F) DoB= 7 years, WoI= 0.74.

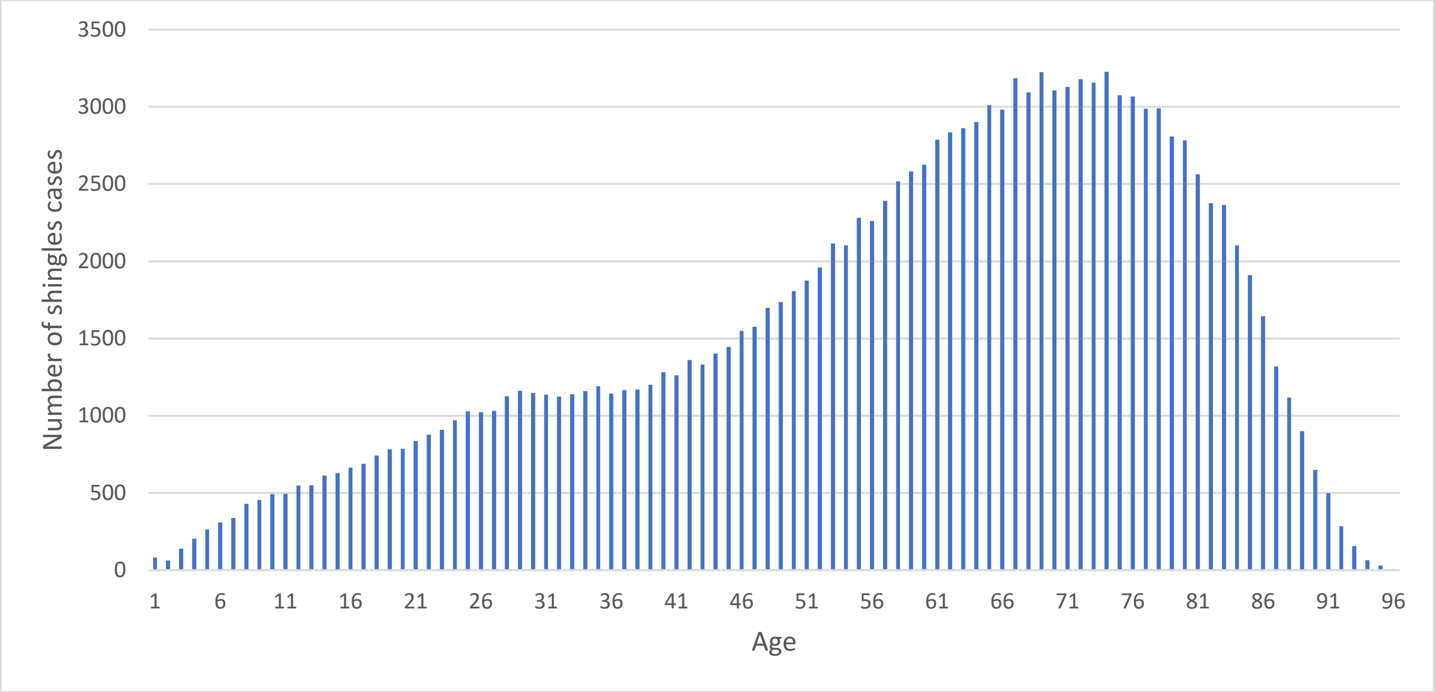

Supplementary Figure 9: Age distribution of shingles cases in baseline scenario.

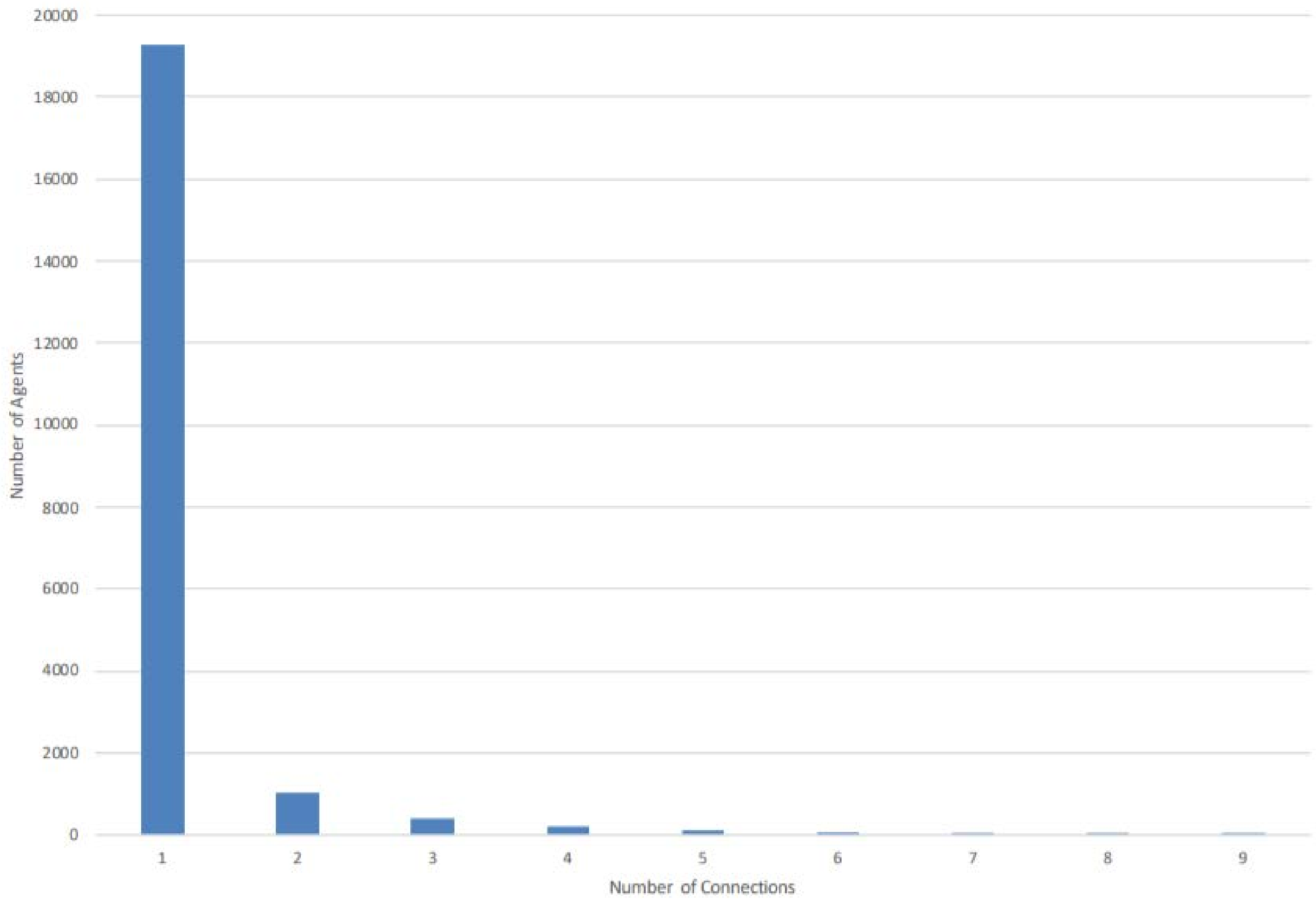

Supplementary Figure 10: Frequency of connections between urban and rural agents. *Y-axis* shows a number of urban agents with one or more connections (*x-axis*) to rural agents in the model. Total population is 483,526.
